# Differential use of nest materials and niche space among avian species within a single ecological community

**DOI:** 10.1002/ece3.70142

**Published:** 2024-09-23

**Authors:** Michael E. Akresh, David Mandell, Peter P. Grima, David I. King, Kathryn Lauer

**Affiliations:** ^1^ Department of Environmental Studies Antioch University New England Keene New Hampshire USA; ^2^ Department of Environmental Conservation University of Massachusetts Amherst Amherst Massachusetts USA; ^3^ Greenfield Massachusetts USA; ^4^ U.S. Forest Service Northern Research Station University of Massachusetts Amherst Amherst Massachusetts USA

**Keywords:** avifauna, niche breadth, niche differentiation, niche segregation

## Abstract

Differential use of resources among bird species has been examined extensively in diet and nesting sites, but few studies have assessed this regarding avian nest materials. We assessed the structure and composition of nests in a group of co‐existing passerine shrubland birds at a site in Massachusetts, USA. We found, measured, collected, and dissected nests, and then weighed nest materials in morphological groups (e.g., bark, twigs, feathers) to determine if our seven focal species were using different nest materials. Among species, we compared proportional material masses in complete nests, and also separately in the exterior, structural part of the nest and the interior, cup lining. We found that the proportional masses of all 17 material types that we examined in nests differed among species. The compositions of nests among all seven bird species were distinct in multivariate ordination space and only a few pairs of species had substantial niche overlap. Proportional masses of materials within discrete sections (exterior and interior) also varied among species. Although some differences in nest composition could be partially explained by bird species size, nest materials differed even within the three larger bodied species and within four smaller bodied species. Our study builds upon previous studies that have shown species‐specificity in avian nest composition and supports the notion that birds using the same environment have distinct niches in relation to the materials placed in their nests. Niche partitioning due to interspecific competition could partially explain our findings, as certain materials are limited as resources, and searching for suitable nest materials is energetically costly. Additionally, other factors, such as partitioned nest sites, may have led to differential nest material use. We recommend further research to help elucidate underlying mechanisms of nest composition partitioning in birds and potentially other nest‐building taxa.

## Graphical abstract

We assessed differential use of nest materials among birds at a site in Massachusetts, USA. Proportional masses of all material types differed among species. Our study supports the notion that birds using the same environment have distinct niches in relation to the materials placed in their nests.

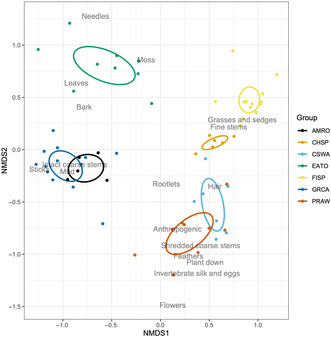

## 1 INTRODUCTION

Co‐existing species within an ecological community often use different resources, such as varied food items or microhabitats, and species correspondingly can have different ecological niches (Da Silva et al., 2017; Teixeira et al., 2021; Ye et al., 2021). A niche can be defined to include the resources that a species utilizes within its biotic and abiotic environment to survive and reproduce (Carscadden et al., 2020; Holt, 1987; Hutchinson, 1957; Schoener, 1974). Species niches may be different and limited resources can be allocated among similar taxa to reduce interspecific competition (Grant & Grant, 1982; Hutchinson, 1959; MacArthur, 1958; Pigot et al., 2018), as postulated by the theory of niche partitioning (also known as niche differentiation, niche segregation, or niche separation). However, additional processes and evolutionary adaptations, unrelated to competition, may also impact resource use and division among co‐existing species (Martin, 1993; Sheard et al., 2023).

With birds, studies have previously examined niche space in a wide variety of forms, including in relation to morphology (Van Valen, 1965), diet (Kent & Sherry, 2020), climate (Ralston et al., 2016), acoustics (Hart et al., 2021), and habitat use (Martin, 1988). For instance, Morimoto and Wasserman (1991) found interspecific differences in singing height and location, foraging height and location, plant species use, and foraging maneuvers among three shrubland bird species in a pine‐barren ecosystem. In terms of habitat use, birds also select different types of nest sites within an ecological community, partitioning available resources such as tree cavities (Schaaf et al., 2020) or other suitable nest locations (Han et al., 2019). Partitioning different nest sites among species can reduce potential predation pressure, as predators may have a more difficult time locating bird nests if they are dispersed across multiple substrates and locations throughout a site (Martin, 1993, 1998).

In addition to preferred nest sites used by a given species, the materials that compose the nest can also represent an “extended phenotype,” unique to a species (Biddle, Broughton et al., 2018; Dickinson et al., 2022). Bird species use a wide variety of nest materials in their nests, including grasses, twigs, hair, feathers, rootlets, and many other materials available to them during nest building (Britt & Deeming, 2011; Hansell, 2000; Reynolds et al., 2016). Nest materials are selected to meet a complex suite of design criteria, providing structural support and insulation for the eggs and young, camouflaging the nest from predators, and protecting the nest from parasites (Biddle et al., 2015; Bailey et al., 2015; Suárez‐Rodríguez et al., 2013). Although nest site variation among species of birds has been studied extensively (Bonaparte & Cockle, 2017; Hanane, 2015; Ye et al., 2019), partitioning of the materials birds use in their nests has rarely been investigated.

Descriptive accounts of nest material variation among bird species have been recorded for decades or even centuries (Audubon, 1844; Bent, 1949; Harrison, 1975; Reed, 1951; Wilson, 1808–1814), but only more recently have researchers examined avian nest materials more quantitatively by examining relative weights of materials within nests (Biddle, Broughton et al., 2018; Crossman et al., 2011). Some recent studies have examined variation in nest composition among species, but these studies have collected nests from different geographic locations (Biddle, Broughton et al., 2018; Dickinson et al., 2022), have examined the use of only certain materials (Deeming, 2018; Dubiec et al., 2013; McFarland & Rimmer, 1996; Surgey et al., 2012), or have focused on only two to three bird species (Alambiaga et al., 2020). Thus, there is limited information on assessing the differential use of nest materials across an avian community at a single site. Also, relatively few studies have actually examined nest materials down to the plant genus level (but see Briggs & Deeming, 2016, 2021), and more research is needed to determine if nest materials vary among species at a fine‐level scale.

We studied nest structure and materials in a co‐existing group of passerine species in a pitch pine‐scrub oak ecosystem in Massachusetts, USA. Our seven focal species overlap breeding territories and co‐exist in shrublands at the site (King et al., 2011). We examined the nest structure, proportion, and weights of materials used among species, and the richness and diversity of materials used, in order to determine if different species were using different materials within the environment. We compared birds of similar size and within family groups to determine the overlap of nest materials used between similar and co‐existing species. Birds often use different materials in the exterior, structural part of the nest versus the inner cup or nest lining (Biddle, Broughton et al., 2018; Deeming, 2023; Dickinson et al., 2022), and we therefore also compared materials across species in each of these nest sections. In addition to broad material types (e.g., leaves, twigs, hair), we also classified graminoids (grasses, sedges, and rushes) to at least the genus level when possible and compared variation within this material type among bird species. We predicted that different species would use different material types and thus have distinct nest composition niches within the same ecosystem.

## 2 METHODS

### 2.1 Study site

As part of ongoing research on shrubland birds in pine barrens (Akresh & King, 2016; Akresh, King, & Marra, 2021; King et al., 2011), we conducted this nest materials study in 2017 and 2018 in the Montague Plains Wildlife Management Area, Massachusetts, USA (42°34′N, 72°31′W). The ~600 ha site is a northeastern USA, pitch pine‐scrub oak barren managed by the Massachusetts Division of Fisheries and Wildlife (see maps in Akresh et al., 2015; Akresh, King, Timm, et al., 2017). We searched for and collected bird nests within shrublands in the site. The surveyed areas had relatively open tree canopies (0–50% canopy cover) dominated by pitch pine (*Pinus rigida*) and tree oaks (*Quercus* spp.). Understory vegetation was a mosaic of dense woody vegetation, some areas of sparse woody vegetation, and patches of herbaceous vegetation with bare mineral soil. Understory woody vegetation included tree saplings (*Acer rubrum*, *Betula populifolia*, *Quercus* spp. and *Prunus pensylvanica*), scrub oaks (*Quercus ilicifolia* and *Q. prinoides*), and other woody plants, such as *Spiraea alba*, *Viburnum cassinoides*, *Prunus susquehanae*, *Corylus cornuta*, *Cornus* spp., and *Vitis* spp. The ground cover included lowbush blueberries (*Vaccinium angustifolium* and *V. pallidum*), *Gaylussacia baccata*, low‐growing *Rubus* spp., graminoids (e.g. *Carex pensylvanica*, *Dichanthelium* spp., *Schizachyrium scoparium*), *Pteridium aquilinum*, and many forbs. Further detailed quantitative information on the vegetation can be found in Akresh (2012), Akresh et al. (2015, 2022), Motzkin (2016), and Motzkin et al. (1996).

### 2.2 Sampling methods

In May and June of each year of the study, we searched for nests of ground and shrub nesting species by conducting systematic searches and observing parental behavior (Martin & Geupel, 1993). We did not monitor all nests to determine nest survival, but instead checked nests 1–7 times after initially finding the nest. After the young fledged or the nest failed (primarily through depredation), we measured the height of the nest in the vegetation (from the ground to the bottom of the nest cup) using a meter stick (±1 cm). We also measured the nest structure while it was still attached to the nest substrate or on the ground. We followed the methods in Akresh, Ardia, and King (2017) to measure nests. Briefly, we used a small ruler (±1 mm) to measure nest width and height, nest cup width and depth, average wall thickness, and floor thickness. We measured nest dimensions (e.g., wall thickness) to where the bulk of the nest material was; we did not include sticks or other materials that were protruding outside of the main nest walls or floor. We calculated cup volume as half of an ellipsoid, using the equation: volume = (4/3π*abc*)/2, where *a* was the cup depth, and *b* and *c* were two cup‐width radii measured perpendicular to each other (Lombardo, 1994). We then carefully removed nests and placed them individually in sealed labeled plastic bags. Nests were then placed in a freezer for at least 3 days to kill any insects and ectoparasites in the nest, and then taken out and allowed to dry for at least 3 days. Nests were stored for 1 week to up to 2 years until dissection.

All nests were measured in the field and after drying were weighed using a digital scale (±0.01 g), and we then dissected a subset of the collected nests to examine nest materials. We found and collected between 10 and 20 nests for eastern towhee, field sparrow, gray catbird, and prairie warbler (scientific names in Table 1), and selected 9–13 nests per species to dissect and examine their nest materials. Nests were randomly selected for dissection; we did not dissect all of the nests due to logistical and time constraints. For American robin, chipping sparrow, and chestnut‐sided warbler, we found 5–7 nests per species, and dissected all of these nests with the exception of one damaged robin nest. We also compiled characteristics of each species, such as the taxonomic family, the range of mass in adult females during the breeding season, the typical nest location, and typical foraging substrate from Birds of the World (2023), augmented by our own field observations of nest locations and heights, and of adults foraging in the study site.

**TABLE 1:** Characteristics of seven avian species which we examined nest dimensions and materials in Montague, Massachusetts, USA.

Species	Family	Mass range (g)	Nest location	Average and SD of nest height (cm)	Foraging substrate
Prairie warbler (*Setophaga discolor*)	Parulidae	7–9	Shrub, sapling	71 (22)	Shrub, tree
Chestnut‐sided warbler (*Setophaga pensylvanica*)	Parulidae	7–9	Shrub, sapling	68 (27)	Shrub, tree
Field sparrow (*Spizella pusilla*)	Emberizidae	11–14	Low vegetation, shrub, ground	32 (27)	Ground, low vegetation
Chipping sparrow (*Spizella passerina*)	Emberizidae	11–15	Shrub, sapling	109 (47)	Ground, low vegetation
Gray catbird (*Dumetella carolinensis*)	Mimidae	32–50	Shrub, sapling	105 (30)	Ground, shrub, tree
Eastern towhee (*Pipilo erythrophthalmus*)	Passerellidae	32–52	Ground, occasional shrub	13 (41)	Ground
American robin (*Turdus migratorius*)	Turdidae	59–91	Shrub, sapling	113 (53)	Ground, shrub, tree

*Note*: Presented are the species name, family, mass range of the adult female, general nest location, average nest height (height above ground) of collected nests in our study site, and typical foraging substrate.

We assessed the type and mass of different materials used to construct the nest by deconstructing the nest and weighing components with a digital scale (±0.01 g; Biddle, Broughton et al., 2018). We separated the nest into exterior vs. interior sections following previous studies (Biddle et al., 2017; Biddle, Broughton et al., 2018; Briggs & Deeming, 2021; Kang et al., 2022). The exterior section consisted of the structural part of the nest (Hansell, 2000), while the interior section was the cup lining and did not contain structural components. Individual material pieces were separated by hand or with forceps into groups of different material types, which were classified into standardized groups based on previous studies (Table 2; Biddle, Broughton et al., 2018; Crossman et al., 2011; Hansell, 2000). Occasionally an individual piece of nest material would be classifiable into multiple types (e.g., a rootlet attached to an above‐ground grass stem), and in these instances, we placed the individual piece into the material group that made up the majority of its mass. After dissecting the nest, we weighed each of the material types separately for each section of the nest. Any masses of materials <0.01 were rounded up to 0.01 g. One American robin nest was fully dissected, but not split into separate nest sections. All dissected nest components were checked for accuracy and consistency. Specifically, material identifications were checked, and any misclassified materials were re‐weighed and material masses changed as needed.

**TABLE 2:** Primary nest material descriptions and classifications used in our study.

Material type	Definition
Anthropogenic	Plastic, string, paper, insulation, and other human‐made materials
Bark	Outer material of woody plants, including grape vines (*Vitis* spp.)
Feathers	All avian feathers
Fine stems	Herbaceous fine stems (<2 mm, not woody or identifiable as grass); generally not shredded
Flowers	Plant flowers, including catkins (e.g., from *Quercus* spp.)
Grasses and sedges	Includes stems, leaves, and reproductive structures of grasses and sedges (Poaceae and Cyperaceae) as well as small amounts of rushes (Juncaceae), exclusive of roots
Hair	Animal hair including horse (*Equus caballus*) hair, deer (*Odocoileus virginianus*) hair, and others
Intact coarse stems	Herbaceous (forbs and ferns), intact (not shredded) stems >2 mm in width and not woody or grass stalks
Invertebrate silk and eggs	Silk from a spider or invertebrate, including any soft insect egg cases
Leaves	All intact or skeletonized leaves including blade and petioles of leaf, generally from a woody plant
Miscellaneous	Pieces too small or deteriorated to identify, and rare materials including snakeskin, acorn pieces, mushrooms, lichen, and seeds
Moss	Mosses (Bryophyta) including moss sporophytes, as well as club mosses (Lycopodiaceae)
Mud	Mud, dirt, and small pebbles
Needles	Needles of conifer species, mostly from pine (*Pinus* spp.), rarely hemlock (*Tsuga canadensis*) or juniper (*Juniperus communis*)
Plant down	Downy/fluffy plant fibers (e.g., from fruits of *Populus* spp. or *Anemone virginiana*)
Rootlets	Roots from woody or herbaceous vegetation
Shredded coarse stems	Herbaceous stems that have been shredded, to create a wispy, thread‐like appearance
Sticks	Woody twigs of any size and width

We additionally sorted and identified plant nest material down to the genus taxonomic level to the best of our ability and weighed these subsets of materials separately. One author (Grima) had extensive expertise with the local flora (Bertin et al., 2020). Grasses (Poaceae) and sedges (Cyperaceae) were particularly common nest components, occurring in the nests of all our focal species. Using reference examples of dominant plant components collected in dormant‐season and remnant conditions from the study site, Grima identified nest materials using recognizable structures, such as grass spikelets, inflorescence architecture, and stem color and texture. On average, we were able to identify 50% of grass and sedge materials (by mass per nest) to genus (SD = 32%, range = 1–100%) in most nests due to intact reproductive structures. The prevalence of identifiable materials in grasses and sedges enabled us to examine their variation among species at a finer taxonomic scale (see below analyses). Botanical nomenclature followed Haines (2011).

### 2.3 Statistical analyses

For each nest, we calculated the proportional mass of the individual material types by dividing the mass of the individual material type by the sum of the masses of the materials. We did not divide by the undissected, total nest mass, because often the weighed total nest mass was slightly higher than the sum of the individual materials, perhaps due to some loss of dust or dirt mass during dissection or for another unknown reason. We also excluded materials that were likely placed or otherwise were present in the nest only after the nest was built, such as insects and nestling fecal sacs (Dickinson et al., 2022). Additionally, we focused our analyses on 17 material types (Table 2) that were on average > 1% of the total nest mass across species (Crossman et al., 2011). The remaining material types (i.e., snake skin, mushrooms, seeds, lichen, and tree sap), and any nest material pieces that were too small to identify, were grouped into the miscellaneous category. Proportional masses of material types were calculated separately for the exterior and interior sections of the nest as well as for the total nest.

To examine differences in material types used among species, we conducted Kruskal–Wallis tests separately for the exterior, interior, and combined sections of the nest. The proportional masses of the individual material types were the response variables, which were often non‐normally distributed, and bird species was our predictor variable. Given that we conducted multiple tests, we adjusted *p*‐values within each nest section analysis set using the Bonferroni‐Holm method (Holm, 1979). All analyses were conducted in R Version 4.2.3 (R Core Team, 2023).

We also calculated the number (richness) of materials in each nest, as well as Simpson's diversity index values from the proportional masses of materials, which effectively examined the richness of materials in each nest and also accounted for the proportional mass of each material. We excluded the miscellaneous/unknown material category before calculating the number of materials and diversity index, as there may have been nest material pieces of already‐documented types in this category that were too small to identify. We compared material richness and diversity among species with Kruskal–Wallis tests.

We also used non‐metric multidimensional scaling (NMDS) to visualize nest material types used among bird species in a multivariate framework. The NMDS used a matrix of nest ID and proportional masses of material types. We removed the miscellaneous material type from the dataset before running the analysis. We chose three dimensions in our NMDS analysis, which yielded a stress value of 0.11. We then plotted the results in ordination plots and presented confidence ellipses of groups of nests per species. The nest material ordination scores outputted from the analysis were also plotted along the new NMDS axes. To statistically test for differences in nest materials among species in a multivariate approach, we conducted an ANOSIM analysis with 9999 permutations. Both the NMDS and ANOSIM were run using the “vegan” package (Oksanen et al., 2022), using Bray‐Curtis ecological distance (Echeverría‐Caro et al., 2024; McGarigal et al., 2000).

To examine niche overlap between pairs of individual species, we calculated the Pianka index using the “spaa” package (Pianka, 1974; Zhang, 2016). We inputted a matrix of the bird species and resources used, specifically the proportional masses of each material type averaged across nests for each bird species. Pianka index values range from 0 to 1 and effectively examine the overlap of resource use, with higher values greater than 0.6 representing an ecologically important overlap between species (Da Silva et al., 2017; Golawski et al., 2020; Kent et al., 2022).

We additionally examined nest composition between the exterior and the interior part of the nest within species. Separately for each species, we used similar multivariate ANOSIM analyses, with Bray‐Curtis ecological distance, to compare the proportional masses (calculated within each section of the nest) of material types between nest sections.

Within grasses and sedges, a material used by all seven focal species, we were interested in comparing variation within this material type among species. For each grass and sedge genus, we calculated the proportional mass, by dividing the mass of the material identified to a given genus by the mass of all the graminoids in a given nest. Using nests with identified graminoids, we compared the proportional masses within genera among bird species with Kruskal–Wallis tests, and adjusted *p*‐values across tests using the Bonferroni‐Holm method. We conducted Kruskal–Wallis analyses only for grass and sedge genera that were identified to be present in at least three nests.

## 3 RESULTS

### 3.1 Nest dimensions and mass

**FIGURE 1:**
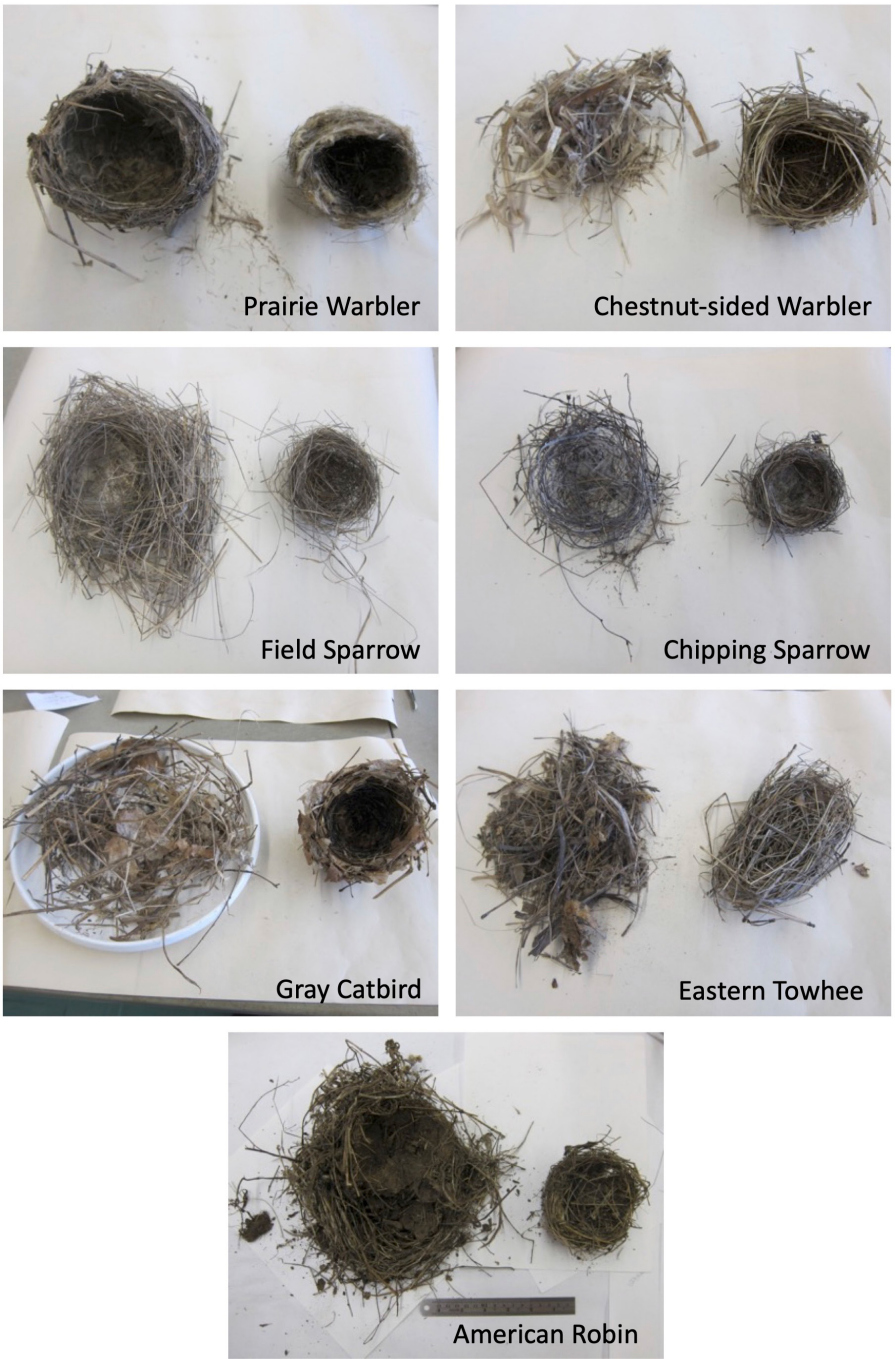
Representative nests of the seven focal species from our study site in Montague, MA, USA, with the exterior (left side) and interior (right side) sections of nests separated. Note the scale is not comparable among species, see Appendix Table for nest dimensions.

Nest dimensions differed among avian species, with smaller species generally building smaller and lighter nests and larger species building larger and heavier nests (Table 1 and Table A1 in Appendix). For example, American robin was the heaviest species examined and had the largest and heaviest nest. However, there was some variation in nest size and mass within bird species of similar mass and family. Within the two species of Emberizidae, chipping sparrows built smaller, lighter, more compact nests compared with field sparrows. Field sparrow nests were often more unkempt, with grass stems sticking out from the nest walls (Figure 1). Eastern towhees, which primarily nest on the ground, had reduced floor thickness, nest height, and nest mass compared with similar‐sized gray catbirds, which primarily place their nests in shrubs. In contrast, within the two species of Parulidae (prairie warblers and chestnut‐sided warblers), nests were of similar size.

### 3.2 Total nest composition

**FIGURE 2:**
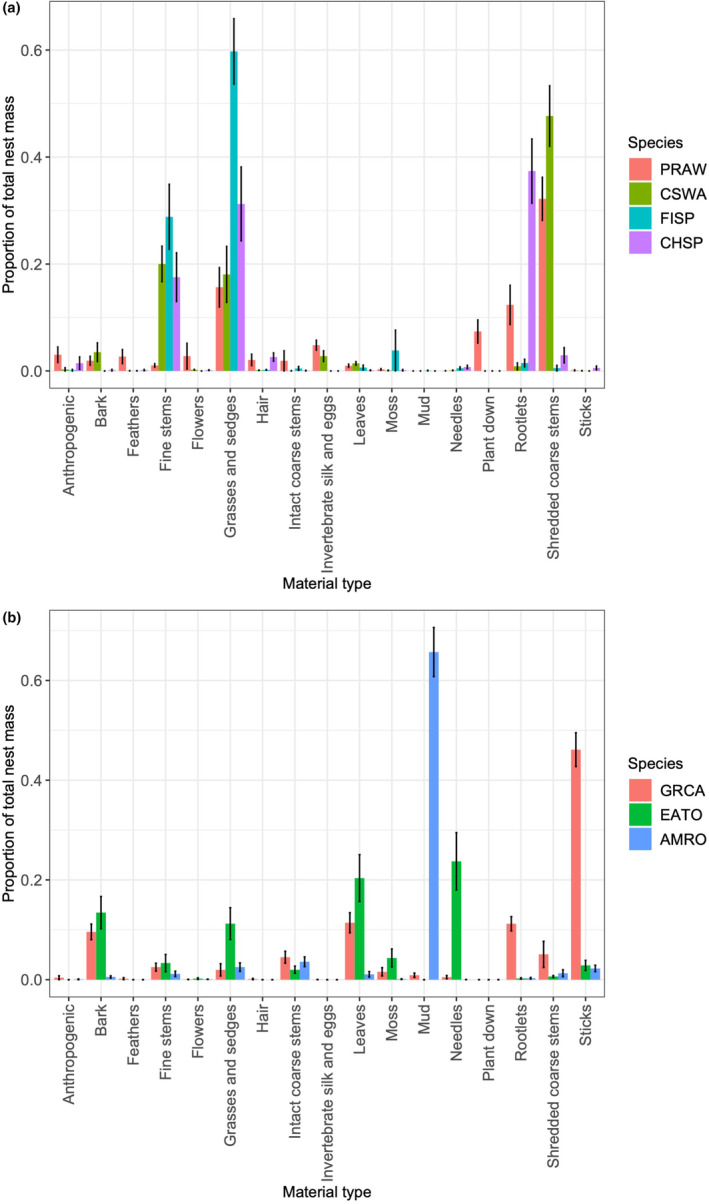
Proportions of different nest material types in the entire nests of (a) the four smaller bodied bird species, and (b) the three larger bodied species, collected from Montague, MA, USA in 2017 and 2018. Bars represent means and error bars are ±1 SE. AMRO, American robin; CHSP, chipping sparrow; CSWA, chestnut‐sided warbler; EATO, eastern towhee; FISP, field sparrow; GRCA, gray catbird; PRAW, prairie warbler.

**FIGURE 3:**
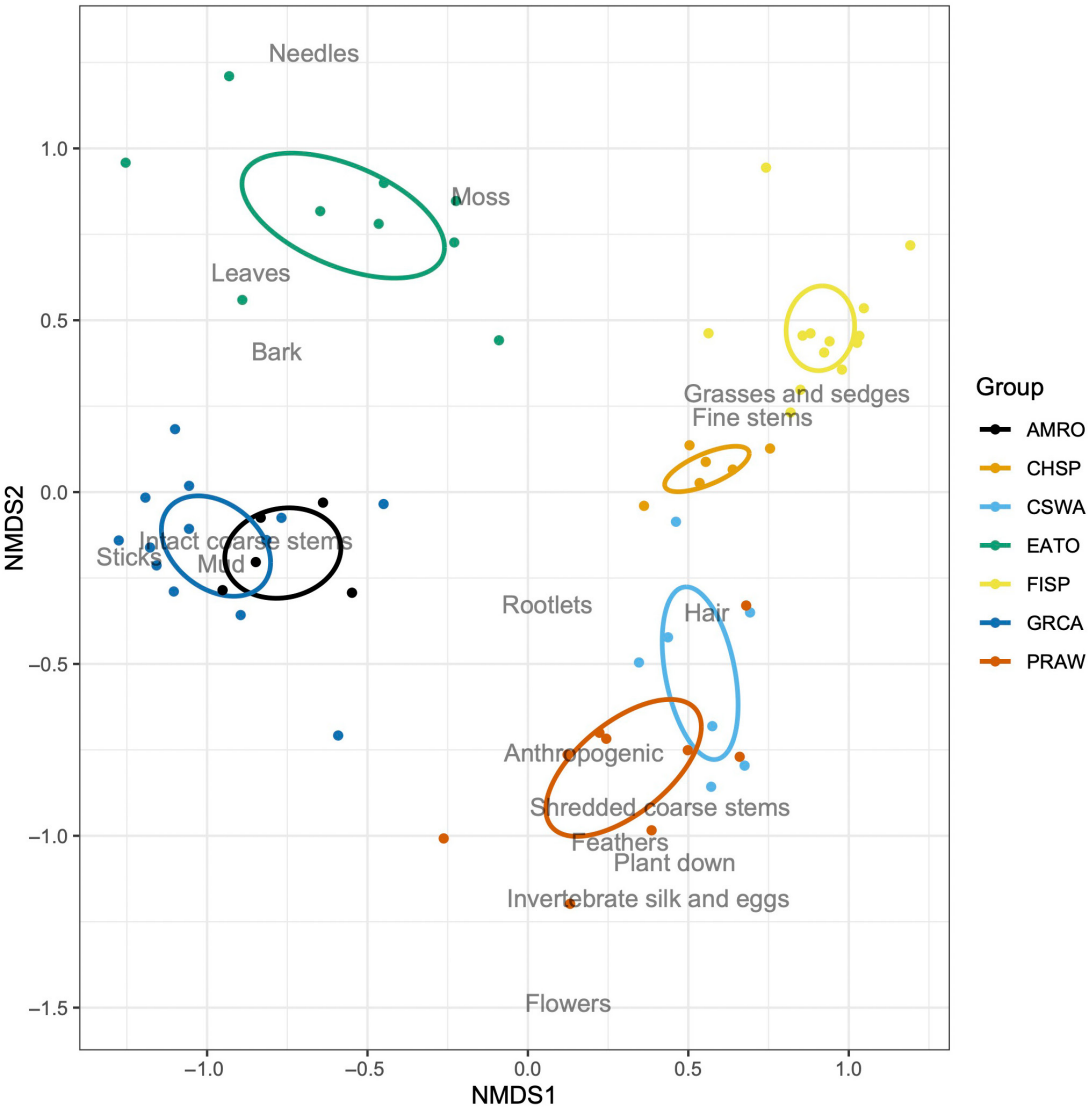
Non‐metric multidimensional scaling (NMDS) of individual nests and their proportional masses of nest material types in ordination space for the first two NMDS axes. Points represent individual nests, and colors represent different bird species. Individual nests closer together have more similar proportional masses of specific nest material types. Also presented are 95% confidence ellipses for nests grouped by bird species. Nest material type labels are plotted from the outputted ordination scores (Table A3), and relate to the nest material types' relationships (strength and direction) along the new NMDS axes. For example, the proportional mass of ‘Flowers’ in a nest is weakly positively associated with the NMDS1 axis, and strongly negatively associated with the NMDS2 axis. Note the labels are slightly ‘jittered’ along the NMDS2 axis to facilitate better viewing.

**FIGURE A1:**
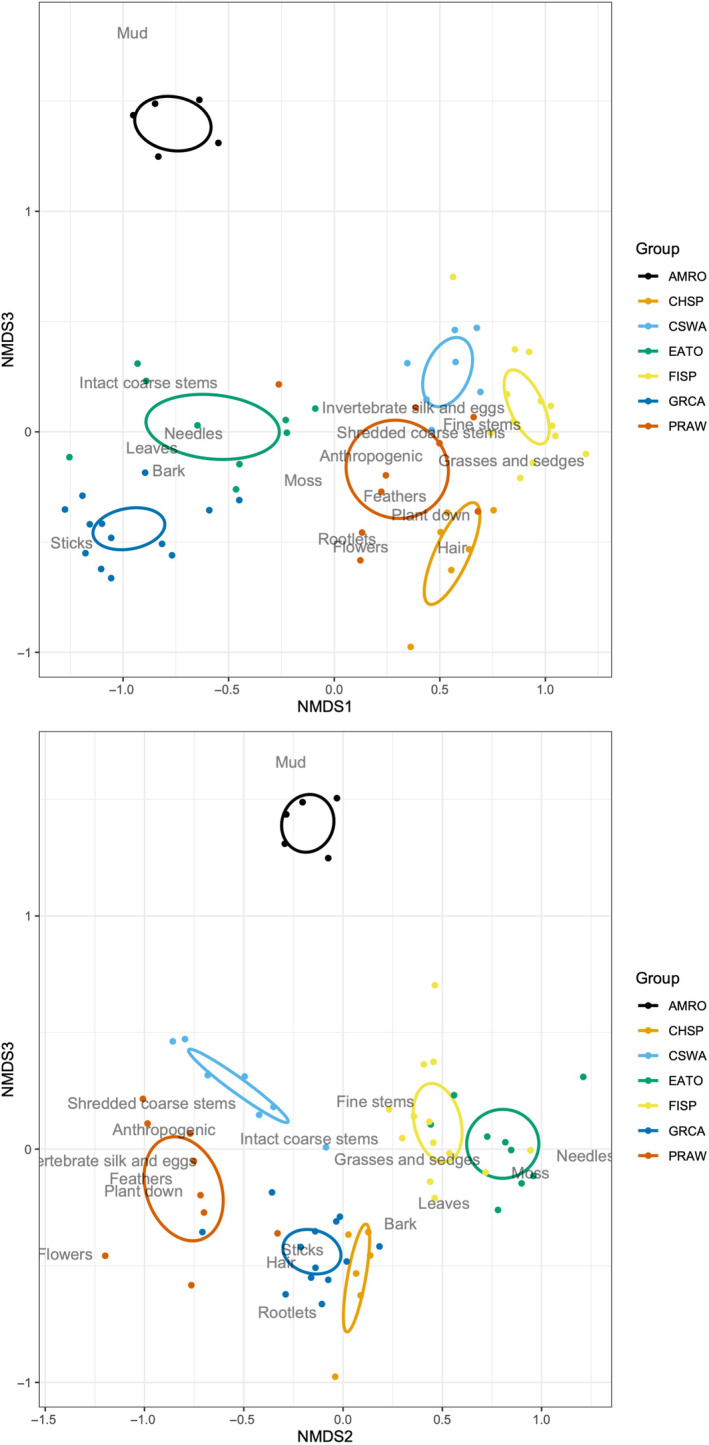
Non‐metric multidimensional scaling (NMDS) of individual nests and nest materials for NMDS axes 1 and 3, and NMDS axes 2 and 3. Points represent individual nests and colors represent different bird species. We also included 95% confidence ellipses. Note the nest material labels are slightly “jittered” to facilitate better viewing.

Material types and quantities differed among species in the entire nest (both sections combined; Figure 2 and Table A2 in Appendix). The proportional masses of all 17 individual material types significantly differed among species when examined separately (Table 3), and combined in a multivariate analysis (ANOSIM: *r* = 0.93, *p* < .001). Additionally, the nest materials differed among almost all of the bird species in ordination space based on non‐overlapping 95% confidence ellipses (Figure 3, Figure A1 and Table A3 in Appendix), although there was some overlap of ellipses between American robins and gray catbird nests as well as between prairie warbler and chestnut‐sided warbler nests for NMDS axes 1 and 2. Examining pairwise niche overlap between species, two pairs had higher overlap in Pianka values (prairie warbler and chestnut‐sided warbler, and field sparrow and chipping sparrow), while all other species pairs had low values <0.6 (indicating non‐overlapping niches; Table 4).

**TABLE 3:** Results of Kruskal–Wallis tests comparing the proportional masses of materials (material mass divided by the total or section nest mass) among our seven focal species.

	Total nest χ^2^	Total nest *p*	Exterior χ^2^	Exterior *p*	Interior χ^2^	Interior *p*
Anthropogenic	20.1	.008	10.6	.10	23.2	.002
Bark	47.5	<.001	32	<.001	44.3	<.001
Feathers	23.4	.003	16.8	.04	30	<.001
Fine stems	38.8	<.001	33.3	<.001	27.4	<.001
Flowers	16.2	.03	14.8	.07	8.4	.21
Grasses and sedges	45.8	<.001	38	<.001	42	<.001
Hair	29.3	<.001	20.1	.01	27.9	<.001
Intact coarse stems	31.7	<.001	33.7	<.001	24.5	.002
Invertebrate silk and eggs	56.4	<.001	55.5	<.001	29	<.001
Leaves	48.2	<.001	42.2	<.001	40.8	<.001
Moss	14.3	.03	13	.09	16.4	.02
Mud	38.6	<.001	39.2	<.001	31.1	<.001
Needles	29.7	<.001	31.6	<.001	34.4	<.001
Plant down	52.2	<.001	34	<.001	47.1	<.001
Rootlets	44.7	<.001	32	<.001	40.9	<.001
Shredded coarse stems	43.1	<.001	41.4	<.001	34.9	<.001
Sticks	53.2	<.001	52.4	<.001	50.1	<.001

*Note*: Separate tests were conducted for each individual material type and for the exterior, interior, and total nest. Presented are the χ^2^ values and adjusted *p*‐values using the Bonferroni‐Holm method (Holm, 1979).

**TABLE 4:** Niche overlap of nest composition between pairs of species, as calculated with Pianka index values using mean proportional masses of nest material types. High values >0.6 are denoted in bold.

	Prairie warbler	Chestnut‐sided warbler	Field sparrow	Chipping sparrow	Gray catbird	Eastern towhee
Chestnut‐sided warbler	**0.87**					
Field sparrow	0.38	0.46				
Chipping sparrow	0.53	0.38	**0.70**			
Gray catbird	0.19	0.14	0.07	0.22		
Eastern towhee	0.18	0.19	0.33	0.24	0.30	
American robin	0.04	0.04	0.04	0.03	0.06	0.03

Of the Parulidae, prairie warblers primarily constructed their nests with shredded coarse stems (e.g., shredded spreading dogbane [*Apocynum androsaemifolium*]), which on average composed of 32% of the nest mass, and they also used sedges, rootlets, plant down, and invertebrate silk, with lesser amounts of anthropogenic material, feathers, bark, catkins, hair, and intact fine and coarse herbaceous stems (Figure 2). Chestnut‐sided warblers also used high proportions of shredded coarse stems (48% of nest mass) and sedges (18%) in their nests, held together with invertebrate silk, but contrastingly used more intact fine herbaceous stems (20%) and bark compared with prairie warblers. Both warbler species' nests were often compact, interwoven cups that were weaved onto the nest substrate with silk and shredded materials (Figure 1).

In contrast to the warblers, almost 60% of field sparrow nest mass consisted of grasses, with another 29% composed of herbaceous fine stems. Chipping sparrow nests were also constructed with different materials compared with the other species, with a much higher proportion of the nest composed of rootlets (37%). Chipping sparrow nests also contained fine stems, grasses, and sedges, similar to the other smaller bodied species, and smaller amounts of shredded coarse stems, hair, anthropogenic material, sticks, and pine needles. Both species of Emberizidae had nests that were placed on top of the nest substrates (rather than being interwoven into the substrate).

The materials used in larger bodied species' nests contrasted greatly with the smaller bodied species, but there was also variation in material types within the larger bodied species. Unlike the other species, gray catbirds built their nests primarily with woody sticks (46% of nest mass), with other materials including rootlets, leaves, and bark. Eastern towhees, primarily a ground‐nesting species, built nests predominantly with pine needles, leaves, bark, and grasses. Lastly, on average, 66% of the American robin nests were comprised of mud, with additional materials including herbaceous stems, grasses, and sticks. Nests of gray catbirds and American robins were built on top of the nest substrate (shrubs or tree branches), while most (90%) of eastern towhee nests were built in a depression on the ground.

**FIGURE 4:**
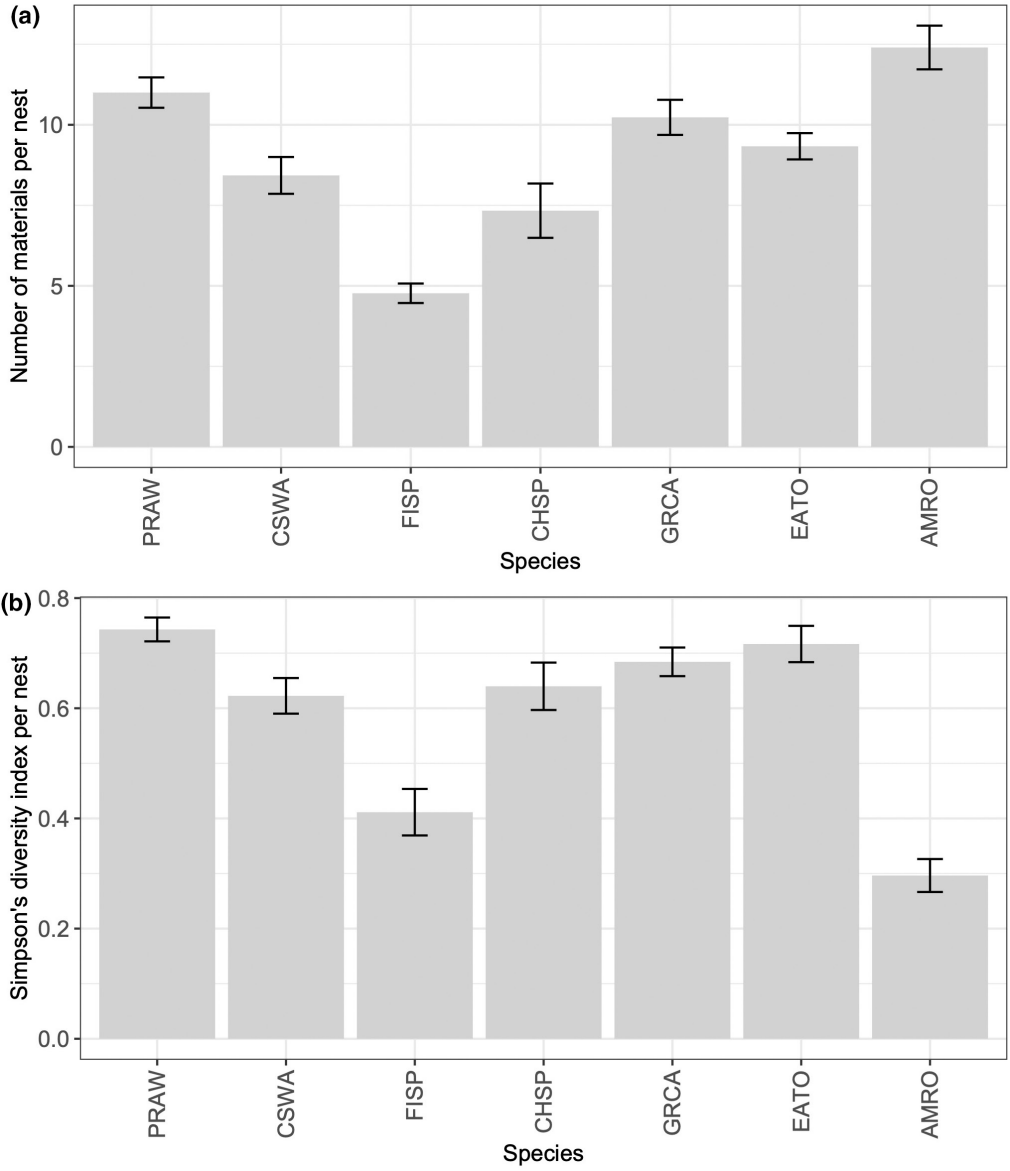
(a) Number of material types (richness), and (b) the Simpson's diversity index for complete nests among species. Bars represent means and error bars are ±1 SE.

The number of materials and Simpson's diversity index of materials differed significantly among species (number of materials: χ^2^ = 42.5, *p* < .001; Simpson's diversity index: χ^2^ = 38.7, *p* < .001; Figure 4). Notably, field sparrows built their nests with fewer material types compared with the other species. Although American robins had the highest number of material types used in their nests of all sampled species, the Simpson's diversity index was the lowest, because the majority of the nest mass was composed of a single material (mud).

### 3.3 Exterior and interior nest composition

**FIGURE 5:**
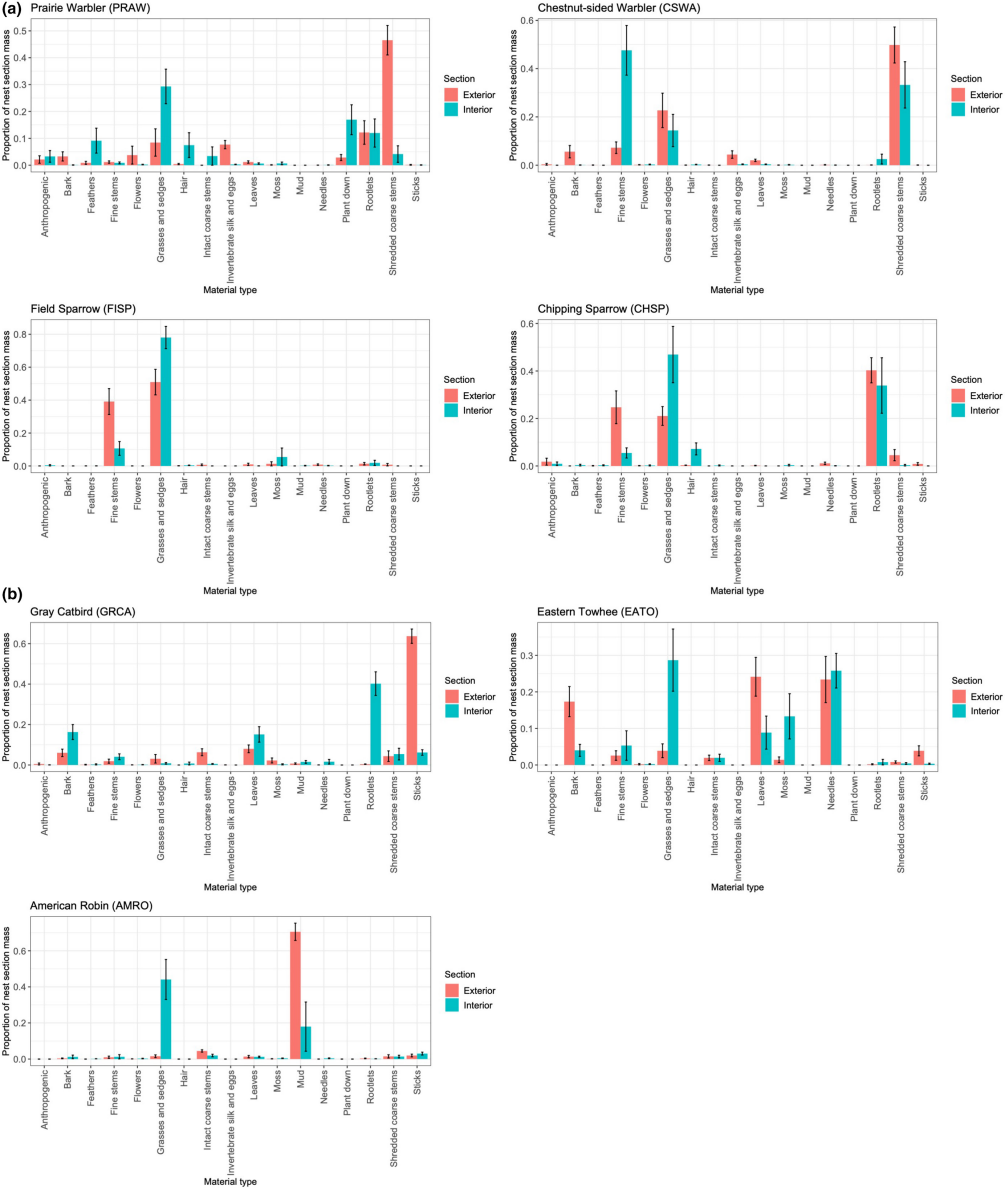
Comparisons of the proportions of nest material types used in the exterior, structural component part of the nest versus in the interior, nest cup, for a) the four smaller species, and b) the three larger species. Bars represent means and error bars are ± 1 SE.

Within species, nest materials were significantly different in the exterior, structural part of the nest compared with the interior, cup of the nest (ANOSIM for PRAW: *r* = 0.65, *p* < .001; CSWA: *r* = 0.43, *p* = .005; FISP: *r* = 0.23, *p* = .004; CHSP: *r* = 0.31, *p* = .01; GRCA: *r* = 0.96, *p* < .001; EATO: *r* = 0.44, *p* = .001; AMRO: *r* = 0.76, *p* = .03). Proportional masses of materials within each nest section also significantly differed among species (Figure 5, Table 3 and Tables A4 and A5 in Appendix). Prairie warbler nests contained relatively more shredded coarse stems in the exterior of the nest and more grasses and sedges, plant down, feathers, and hair in the interior of the nest. For chestnut‐sided warblers, bark was primarily used in the exterior of the nest, while fine stems were much more prevalent in the interior. In contrast, chipping sparrows used more fine stems in their exterior nest sections, and more grasses in the interior sections. Within species, certain materials were sometimes used in similar proportions in both exterior and interior sections, such as rootlets in prairie warbler and chipping sparrow nests. Field sparrows used grasses extensively in both sections of the nest, with larger, wider grass pieces on the exterior and smaller, thinner pieces on the interior (Figure 1).

Larger bodied species often used different materials in their exterior and structural part of the nest compared with the smaller bodied species. Gray catbirds used primarily sticks, American robins used mud, and eastern towhees placed bark, leaves, and needles in their exterior nest sections. Gray catbird nest interiors were formed with a cup of intertwining rootlets, though there was also often an intermediate, middle layer of leaves and bark (which was partitioned to the exterior/structural section). American robin and eastern towhee nest interiors were usually composed of grass and other unidentified material, while eastern towhees also had pine needles, moss sporophytes, leaves, and bark in their interior cups.

### 3.4 Variation in grass and sedge genera among bird species

**FIGURE 6:**
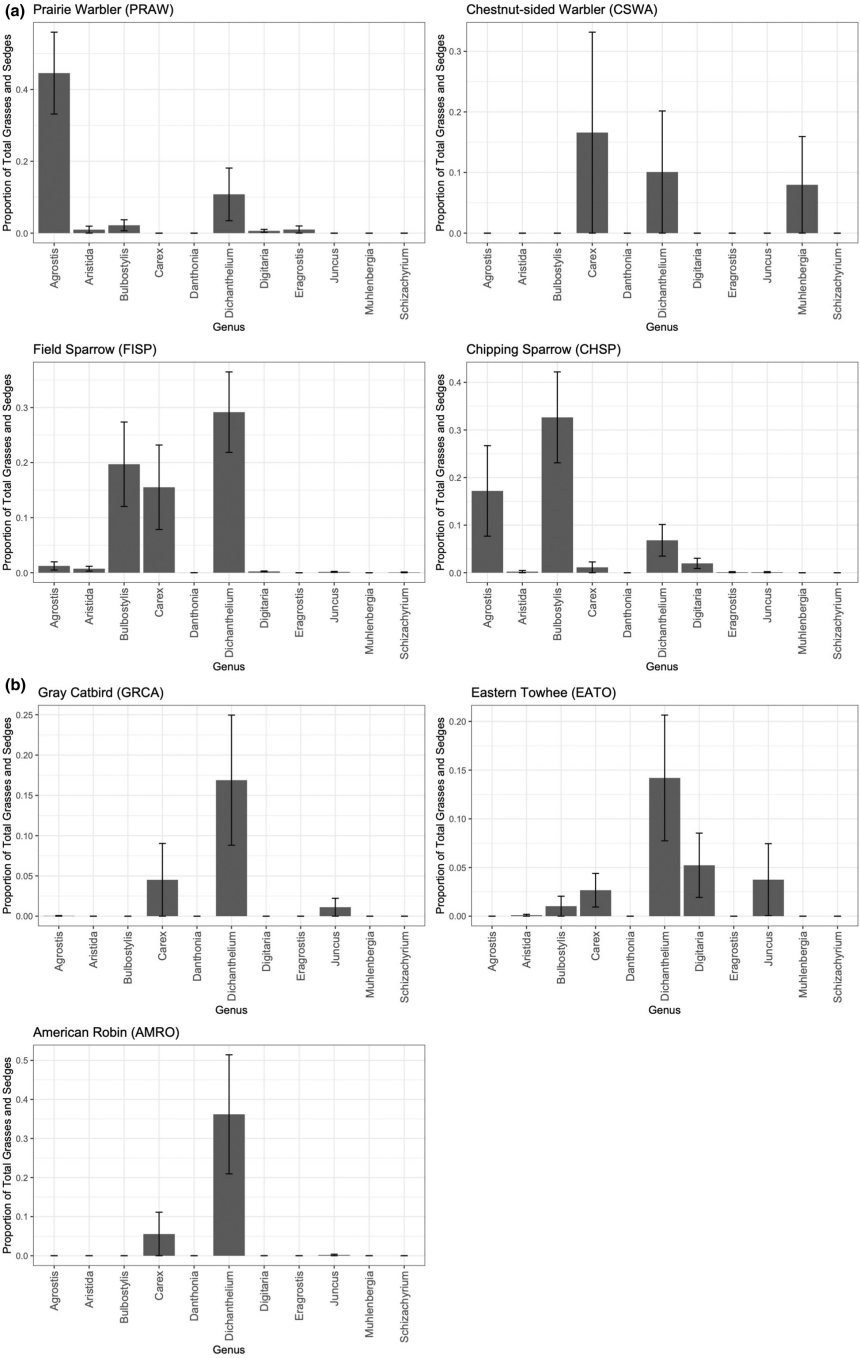
Proportional masses of grass and sedge genera (identified mass divided by the total mass of grasses and sedges in nests) of a) the four smaller‐bodied bird species, and b) the three larger‐bodied species, collected from Montague, MA, USA in 2017 and 2018. Bars represent means and error bars are ± 1 SE.

The proportional mass of identified grasses or sedges within two genera, *Agrostis* and *Bulbostylis*, varied significantly among bird species (*Agrostis*: χ^2^ = 25.2, adjusted *p* = .002; *Bulbostylis*: χ^2^ = 26.6, adjusted *p* = .001, Figure 6 and Table A6 in Appendix). Prairie warblers and chipping sparrows used relatively more *Agrostis* in their nests compared with the other species. Additionally, field sparrows and chipping sparrows used relatively more *Bulbostylis* in their nests. We did not detect significant differences among bird species in four other grass and sedge genera (*Aristida, Carex, Dichanthelium*, and *Digitaria*; all had adjusted *p* > .43); notably, *Dichanthelium* was placed in the nests of all seven bird species (Figure 6).

## 4 DISCUSSION

Our study documented differences in nest structure and composition among seven passerine species co‐existing within a single site. Our results are consistent with other studies that have examined nests of fewer species of birds at single locations and found interspecific differences in nest composition (Alambiaga et al., 2020; Crossman et al., 2011). Several different mechanisms for our findings are possible and may be co‐occurring, including nest material variation due to the bird species' ecology (nest site, size, and mass of the species) and because of interspecific competition for materials.

The use of different nest sites among co‐occurring passerines is well documented (Han et al., 2019; Martin, 1998), and species could also be co‐evolving to partition available nest materials to best camouflage their nests in their preferred nesting sites (Bailey et al., 2015; Hansell, 1996). We observed ground‐nesting eastern towhees had high proportions of pine needles, leaves, and grasses in their nests, which can readily blend in with the surrounding ground cover and debris in our pitch pine‐scrub oak barrens study site. Gray catbirds nested in dense shrubs, and the woody sticks used in the outer portion of their nest may help the nest camouflage with its surroundings. Field sparrows nested fairly low to the ground (often <1 m in height), occasionally near clumps of sedges and grasses, concealing their grass‐based nest. Lastly, many of the chipping sparrow nests were built within pitch pine saplings, and the dark rootlets in the exterior of the nests blended in with the adjacent pitch pine bark (MA personal observation). Material partitioning could therefore be due to adaptations related to preferred nest sites and pressures of nest depredation by predators (Martin, 1993).

A non‐mutually exclusive hypothesis for differences in avian nest composition could be niche partitioning of nest materials, in which birds co‐occurring in the same habitat have adapted to reduce competition for nest materials by selecting different material types (Schoener, 1974). For interspecific competition to occur, the resource must be limited (e.g., the use of certain nesting material would decrease its availability for others), co‐existing species compete for the resource (e.g., multiple species overlap in the use of a given nest material type), and reduced availability of the resource has fitness consequences (Dhondt, 2012, Kent et al., 2022). At least some nest materials are limited in the environment; for example, feathers are often in short supply and are highly sought after by birds as nesting material because they can provide insulation or other fitness benefits to nestlings (Hansell, 1995; Ruiz‐Castellano et al., 2018; Windsor et al., 2013; Wolfe‐Merritt et al., 2022). Other limited materials likely include fresh invertebrate silk (Low et al., 2013) and animal hair within the 0.5–2 ha home ranges of small passerine birds (Akresh et al., 2015; Billerman et al., 2023). Even more abundant materials such as exposed rootlets or certain types of grasses or forbs are patchily spaced throughout a landscape and could be limited adjacent to a given nest site. Although passerine birds may travel relatively far distances such as 500 m to find nest materials (Surgey et al., 2012), most collecting trips are often much shorter distances from the nest (Rydgren et al., 2023; Wesołowski & Wierzcholska, 2018). For instance, 75% of collection trips for the exterior section of prairie warbler nests were within 25–30 m of the nest, in a population studied in Indiana (Nolan Jr., 1978).

We demonstrate that co‐existing species do use some of the same materials (e.g., bark, grasses, rootlets, fine stems) in their nests, albeit at different proportions, and thus may compete for them. Although the establishment of breeding territories can mediate within‐species competition for nest materials, individuals of different passerine bird species overlap territories within a shared area (King et al., 2011; Willson, 1974), giving rise to potential competition and specialization (Hansell, 1995). Intraspecific competition and stealing existing nest materials from others' nests have been documented in over 10 colonial nesting species (Hansell, 2000; Moreno et al., 1995). At least one study (Smith, 1980) has recorded interspecific stealing of nest materials, specifically grasses, in five species of Tyrannidae. Further research would be useful to determine the existence of additional direct competition for nest materials across species. In addition to direct competition, our focal co‐existing shrubland bird species use similar open‐canopy habitats throughout their ranges (Akresh, King, Lott et al., 2021; Schlossberg & King, 2007), and may have competed for nest materials in their evolutionary history, co‐evolving to partition different material types over time (the ‘ghost of competition past’ theory; Connell, 1980; Ye et al., 2019).

The final requirement for an interspecific competition hypothesis would be potential fitness consequences. Building nests and searching for materials is energy and time‐intensive (Bailey et al., 2016; Lens et al., 1994; Mainwaring & Hartley, 2009, 2013; Moreno et al., 2010). Niche partitioning of nest materials could reduce energy expenditure, search distances, and search times for materials in co‐existing species with overlapping home ranges (Surgey et al., 2012), theoretically improving the fitness of co‐existing species. Passerine birds make hundreds of trips to find materials and build their nests over the course of several days (Sturm, 1945; Stanley, 2002; Nolan Jr., 1978), and thus minimizing time and distance to find materials could decrease energy expenditure and stress levels (Collias & Collias, 1984; Moreno et al., 2008). Prairie warblers and other species have also been observed to reuse nest materials from failed nests (Hebda, 2007; Nolan Jr., 1978; Sturm, 1945, MA personal observations), highlighting the cost of finding new materials. Reducing the nest‐building time between successive nesting attempts during short breeding seasons could increase fitness by allowing more time for renesting after nest failures (Morrison et al., 2019), or by producing earlier‐fledging young that obtain better quality wintering habitat (Akresh, King, & Marra, 2021) and have increased survival (McKim‐Louder et al., 2013).

Nevertheless, other hypotheses for why bird species differ in their nest compositions are also possible, such as due to nest structure, the size and mass of the bird, bill shape and length, foraging behavior, and availability of nesting material in the given habitat type (Deeming, 2023; Hansell, 2000). Some interspecific differences are likely due to specific structural components of a particular bird species' nest. For instance, field sparrows may use certain grasses and stems to create the interlocking structural support framework in their nests, while warblers may use certain materials that are best able to interconnect with a “velcro” attachment to spiderweb and silk (Hansell, 2000). We also observed different materials being used between larger‐ and smaller bodied species (and correspondingly larger and smaller nests), though within similar‐sized species (e.g., gray catbird versus eastern towhee), there existed differentiation in nest materials that were not due to species' size. A recent broad‐scale analysis across avian species also showed some support for nest material differentiation due to body mass and beak morphology, although there was still considerable variation (~50%) unrelated to these factors (Sheard et al., 2023). Similarly, we also found higher niche overlap of nest materials between bird species with similar morphologies and in the same family (within Parulidae and Emberizidae), but some differences still existed in nest composition within families. Lastly, some studies have noted different materials used in different geographic locations, latitudes, or local habitats (Briggs & Deeming, 2021; Briggs et al., 2019; Crossman et al., 2011). In our study, nests of the focal species were interspersed throughout a single site and multiple species nested adjacent to each other (e.g., within 50 m). We suspect that microhabitat and vegetation composition around each nest could have explained some variation of nest materials within bird species (and variation of the different plant genera used within the broad material types), but microhabitat was likely less important in nest composition variation among species. As more studies document nest materials across species worldwide, further research on body size, bill size and shape, material availability, taxonomic relationships, geographic location, and other factors in relation to nest material differentiation would be important to better understand avian evolution and biology.

Additional species co‐exist in the shrubland habitats at our study site, including black‐capped chickadees (*Poecile atricapillus*), pine warblers (*Setophaga pinus*), black‐and‐white warblers (*Mniotilta varia*), common yellowthroats (*Geothlypis trichas*), and brown thrashers (*Toxostoma rufum*; King et al., 2011). Although we did not examine nests of these other species (including cavity‐nesters, tree‐nesters, or otherwise nests of species that were very difficult to find or access), past qualitative examinations of nest materials show that at least some of these other species likely use different primary material types in their nests compared with our focal species (Billerman et al., 2023). For example, black‐capped chickadees line their nest cavities primarily with green moss and hair (Harrison, 1975), two material types that were not used extensively by our focal species.

Birds may also be using specific materials non‐randomly even for more common material types (Briggs & Mainwaring, 2019; Briggs et al., 2019). Grasses and sedges were used across all species, but some bird species used particular types of grasses (e.g., field sparrow nests had more dried pieces of *Bulbostylis capillaris*). Our results are consistent with Wesołowski and Wierzcholska (2018), a study which also examined interspecific variation within a nest material type, as they found different species of tits differentiating and selecting to use, to some degree, different bryophyte species for their nests. Besides partitioning different grass and sedge genera, larger bodied birds in our study often used larger and longer pieces of grass stems in their nests, especially in the exterior section, while the warbler species often used shorter, smaller, and more flexible pieces (MA personal observation).

Our novel, quantitative examination of nest materials for our seven focal species was similar to nest compositions observed in qualitative assessments by other studies elsewhere (Billerman et al., 2023; Harrison, 1975). For instance, American robin nests are ubiquitous and relatively easy to find in suburban settings, and it is well known for centuries that this species uses mud, grass, and woody sticks in their nest (Audubon, 1844; Billerman et al., 2023). Nevertheless, our study is one of the first to quantitatively separate and weigh each of the nest material types and present values for our focal species that can be compared with other species worldwide (Deeming & Mainwaring, 2015; Dickinson et al., 2022). In Europe, nests of the Turdidae family also have high proportional masses of mud, and nests of Emberizidae also have high proportions of grasses (Deeming, 2023). However, to date, nests of less than 1% of all passerine species have been quantitatively examined with dissected materials separated and weighed (Deeming, 2023; Dickinson et al., 2022).

We acknowledge that there is some inherent bias with our (and other studies') weighing method, as lighter materials such as hair and feathers may tally to hundreds of pieces and comprise a significant proportion of the physical volume of the nest walls or lining, but may not comprise a high proportion of nest mass if other, heavier materials are present (Elts, 2005). An alternative, previously conducted approach has been to manually count the number of sticks or feathers in a given nest (e.g., Kern, 1984; Nolan Jr., 1978; Stanley, 2002). Although this alternative method may be more suitable for lighter materials, this counting method is especially time‐consuming, and the relative proportional space that the materials are taking up in the nest is dependent on the length and width of the material pieces. Some types of lighter materials, such as plant down, would also not be possible to count or measure their number or length quantitatively. Further research and consideration of different quantitative methods (such as the proportional volume of materials) to determine nest composition would be useful, as more studies on nest composition and more data becomes available on species throughout the world.

Consistent with other studies, we found birds in our study site often use different materials in the exterior and structural part of the nest compared with the interior, cup lining (Briggs & Deeming, 2021; Biddle, Deeming, & Goodman, 2018; Hansell, 2000), and this also varied by species. Large‐bodied species need relatively more support for larger nestlings and adults in the nest (Biddle et al., 2015; Deeming, 2018), and in American robins and gray catbirds, mud and woody sticks provide that needed structure (Biddle et al., 2017). Eastern towhees, despite being large‐bodied, nested primarily on the ground, and thus non‐rigid materials, such as pine needles, leaves, and grasses in the exterior of their nests may be used because the ground is already providing the structure. The one towhee nest we found above ground and dissected was also comprised of the same materials; thus, presumably enough leaves and bark can still provide the needed support for this species. In all our focal species, finer, often softer materials were used in the interior and cup lining of the nest, such as fine stems, grasses, hair, feathers, or fine rootlets. Some of these materials such as hair and feathers could be providing thermal insulation (Hilton et al., 2004). However, nest insulation may not influence reproductive success in open‐cup passerines (Akresh, Ardia & King, 2017), and hair may take longer to dry out and lose insulating capacities after being wet (Biddle et al., 2019; Deeming & Campion, 2018). Alternatively, finer materials could primarily be providing softer, more padded material for the fragile eggs and nestlings (Biddle, Broughton et al., 2018), or contributing more flexible materials that can expand as nestlings grow in the nest (Deeming, 2023; Slagsvold, 1989).

Six of the seven focal species had some anthropogenic material placed in their nests and amounts varied among species. Anthropogenic materials in the nests we examined, such as wide and narrow strips of plastic, and non‐natural insulation/filling (polyester or polyurethane fibers), may resemble and are likely being used as replacements for natural materials with similar consistencies (e.g., leaves, shredded herbaceous stems, and plant fluffy material, respectively; Collias & Collias, 1984; Jagiello et al., 2023). Numerous other studies have also noted the use of anthropogenic materials in passerine nests, often in increased abundance in more urban or agricultural areas (Antczak et al., 2010; Townsend & Barker, 2014; Vasquez et al., 2022; Wang et al., 2009). Although our study site was situated in a natural environment, there are roads within and adjacent to the site (Akresh & King, 2016), and some illegal dumping of refuse has occurred along roads in the past. Indeed, bird nests can be used to quantify the presence and abundance of plastics or other non‐natural waste materials at a given spatial location or time (Blettler et al., 2020; Briggs et al., 2023; Potvin et al., 2021), but as we found, some species may use anthropogenic materials more so than others.

In conclusion, our study shows that individual species use different materials in their nests within a single site, and provides a basis for further research examining various hypotheses for our results, such as the theory of niche partitioning. Differentiation of nest materials may also have co‐evolved with nest site partitioning (Han et al., 2019; Martin, 1993), as birds use specific materials to better conceal their nests and support the nestlings on a given nest substrate. Our novel quantitative examination of materials in the exterior and interior sections, and complete nests of a set of co‐existing species, along with further research, are important for better understanding the ecology and evolution of birds and other taxa that build nests.

## AUTHOR CONTRIBUTIONS

**Michael E. Akresh:** Conceptualization (lead); data curation (equal); formal analysis (equal); funding acquisition (equal); investigation (lead); methodology (lead); project administration (lead); resources (equal); supervision (equal); validation (equal); visualization (equal); writing – original draft (lead); writing – review and editing (lead). **David Mandell:** Data curation (supporting); formal analysis (equal); investigation (equal); validation (supporting); visualization (equal); writing – original draft (supporting); writing – review and editing (equal). **Peter P. Grima:** Data curation (equal); investigation (equal); methodology (supporting); validation (equal); writing – review and editing (equal). **David I. King:** Funding acquisition (equal); investigation (supporting); project administration (equal); resources (equal); supervision (equal); writing – review and editing (equal). **Kathryn Lauer:** Data curation (supporting); investigation (equal); visualization (supporting); writing – review and editing (supporting).

## FUNDING INFORMATION

Funding was provided by the US Forest Service Northern Research Station as part of a larger project. Additional funding came from Antioch University New England.

## CONFLICT OF INTEREST STATEMENT

The authors declare no conflict of interest.

## Data Availability

Data are available from the Dryad Digital Repository: https://doi.org/10.5061/dryad.1jwstqk41.

## APPENDIX 1

**TABLE A1:** Dimensions of nests in seven species found in Montague, MA, USA.

	*n*	Nest height (mm)	Nest diameter (mm)	Cup diameter (mm)	Cup depth (mm)	Wall thickness (mm)	Floor thickness (mm)	Nest cup volume (cm^3^)	Mass (g)
Prairie warbler	20	62 (6)	70 (5)	42 (4)	37 (5)	14 (3)	25 (7)	34 (8)	6.06 (1.34)
Chestnut‐sided warbler	7	67 (15)	78 (6)	49 (3)	36 (6)	14 (2)	31 (11)	46 (11)	5.09 (1.36)
Field sparrow	18	59 (11)	89 (6)	52 (5)	44 (10)	18 (3)	16 (8)	61 (19)	6.75 (1.94)
Chipping sparrow	5	49 (1)	74 (5)	42 (8)	36 (5)	14 (5)	13 (5)	34 (16)	4.36 (1.59)
Gray catbird	19	89 (16)	132 (10)	82 (7)	50 (7)	26 (6)	39 (14)	179 (48)	66.12 (17.95)
Eastern towhee	10	73 (19)	129 (8)	80 (7)	46 (13)	25 (4)	28 (19)	150 (50)	26.48 (13.74)
American robin	6	102 (22)	131 (7)	95 (8)	58 (8)	18 (4)	44 (18)	271 (48)	206.78 (36.40)

*Note*: Presented are means with SD in parentheses.

**TABLE A2:** Raw masses (g) of nest material in complete nests (both sections combined) among our seven focal species for the dissected nests.

	Prairie warbler	Chestnut‐sided warbler	Field sparrow	Chipping sparrow	Gray catbird	Eastern towhee	American robin
Sample size	9	7	13	6	13	9	5
Anthropogenic	0.208 (0.315)	0.009 (0.023)	0.008 (0.031)	0.085 (0.172)	0.26 (0.899)	0 (0)	0.144 (0.322)
Bark	0.121 (0.165)	0.181 (0.222)	0 (0)	0.008 (0.02)	6.018 (3.446)	3.153 (2.093)	1.174 (0.997)
Feathers	0.196 (0.322)	0.001 (0.004)	0.002 (0.006)	0.005 (0.008)	0.119 (0.418)	0 (0)	0.01 (0.007)
Fine stems	0.067 (0.069)	0.927 (0.309)	1.84 (1.45)	0.732 (0.568)	1.555 (1.644)	1.053 (1.686)	2.504 (2.64)
Flowers	0.147 (0.37)	0.01 (0.008)	0.001 (0.003)	0.005 (0.005)	0.036 (0.058)	0.109 (0.276)	0.2 (0.174)
Grasses and sedges	0.98 (0.809)	1.024 (0.906)	4.006 (2.283)	1.14 (0.547)	0.941 (1.835)	3.172 (3.566)	5.574 (4.345)
Hair	0.132 (0.23)	0.007 (0.008)	0.012 (0.019)	0.118 (0.108)	0.081 (0.282)	0 (0)	0 (0)
Intact coarse stems	0.129 (0.387)	0.001 (0.004)	0.042 (0.127)	0.002 (0.004)	3.264 (3.93)	0.502 (0.508)	7.896 (4.692)
Invertebrate silk and eggs	0.282 (0.165)	0.124 (0.115)	0 (0)	0 (0)	0.015 (0.055)	0 (0)	0 (0)
Leaves	0.062 (0.063)	0.074 (0.057)	0.033 (0.076)	0.003 (0.005)	6.821 (3.589)	5.586 (4.751)	2.368 (2.626)
Miscellaneous	0.662 (0.442)	0.281 (0.276)	0.232 (0.255)	0.178 (0.073)	2.502 (1.658)	5.519 (6.657)	44.464 (24.181)
Moss	0.017 (0.027)	0.004 (0.008)	0.143 (0.513)	0.008 (0.02)	0.952 (1.708)	1.067 (1.183)	0.276 (0.354)
Mud	0 (0)	0 (0)	0.003 (0.011)	0 (0)	0.562 (0.966)	0 (0)	142.48 (32.121)
Needles	0.002 (0.007)	0.006 (0.01)	0.029 (0.053)	0.033 (0.04)	0.397 (1.081)	5.604 (3.929)	0.06 (0.08)
Plant down	0.422 (0.353)	0 (0)	0 (0)	0 (0)	0.001 (0.003)	0 (0)	0.01 (0.022)
Rootlets	0.743 (0.628)	0.053 (0.107)	0.118 (0.23)	1.485 (0.741)	6.91 (3.401)	0.098 (0.209)	0.632 (0.729)
Shredded coarse stems	1.977 (0.956)	2.254 (0.685)	0.052 (0.186)	0.082 (0.095)	2.518 (4.223)	0.176 (0.15)	2.896 (3.58)
Sticks	0.007 (0.017)	0.001 (0.004)	0 (0)	0.025 (0.042)	30.23 (13.339)	0.769 (0.792)	4.706 (2.783)

*Note*: Presented are means and SDs in parentheses.

**TABLE A3:** Non‐metric multidimensional scaling (NMDS) ordination scores of nest material types for each of the three NMDS axes.

Material type	NMDS1	NMDS2	NMDS3
Anthropogenic	0.174	−0.875	−0.063
Bark	−0.782	0.274	−0.222
Feathers	0.288	−1.066	−0.197
Fine stems	0.700	0.183	0.165
Flowers	0.126	−1.359	−0.417
Grasses and sedges	0.839	0.346	−0.047
Hair	0.558	−0.361	−0.497
Intact coarse stems	−0.878	−0.165	0.087
Invertebrate silk and eggs	0.375	−1.173	−0.031
Leaves	−0.862	0.541	−0.208
Moss	−0.146	0.968	−0.069
Mud	−0.955	−0.240	1.745
Needles	−0.666	1.225	0.086
Plant down	0.459	−1.048	−0.24
Rootlets	0.061	−0.274	−0.628
Shredded coarse stems	0.412	−0.914	0.091
Sticks	−1.242	−0.171	−0.515

**TABLE A4:** Raw masses (g) of nest material in the exterior section of the nest among our seven focal species.

	Prairie warbler	Chestnut‐sided warbler	Field sparrow	Chipping sparrow	Gray catbird	Eastern towhee	American robin
Sample size	9	7	13	6	13	9	4
Anthropogenic	0.096 (0.229)	0.009 (0.023)	0 (0)	0.063 (0.128)	0.25 (0.901)	0 (0)	0 (0)
Bark	0.12 (0.166)	0.18 (0.222)	0 (0)	0 (0)	3.051 (3.451)	2.909 (1.923)	0.84 (0.921)
Feathers	0.05 (0.11)	0.001 (0.004)	0.001 (0.003)	0.002 (0.004)	0.056 (0.199)	0 (0)	0.002 (0.005)
Fine stems	0.044 (0.054)	0.214 (0.178)	1.609 (1.261)	0.672 (0.54)	0.781 (1.315)	0.68 (1.213)	2.143 (2.751)
Flowers	0.142 (0.367)	0.006 (0.005)	0.001 (0.003)	0.003 (0.005)	0.02 (0.041)	0.09 (0.251)	0.228 (0.167)
Grasses and sedges	0.436 (0.927)	0.71 (0.682)	2.198 (1.855)	0.492 (0.264)	0.766 (1.57)	1.28 (2.743)	3.452 (3.433)
Hair	0.019 (0.032)	0.001 (0.004)	0.004 (0.008)	0.008 (0.004)	0.001 (0.003)	0 (0)	0 (0)
Intact coarse stems	0 (0)	0.001 (0.004)	0.042 (0.127)	0 (0)	3.143 (3.818)	0.378 (0.414)	9.62 (2.449)
Invertebrate silk and eggs	0.277 (0.161)	0.117 (0.11)	0 (0)	0 (0)	0.015 (0.055)	0 (0)	0 (0)
Leaves	0.052 (0.062)	0.069 (0.056)	0.032 (0.075)	0.003 (0.005)	3.671 (3.347)	4.98 (4.388)	2.865 (2.596)
Miscellaneous	0.36 (0.161)	0.267 (0.263)	0.164 (0.178)	0.117 (0.067)	1.229 (0.835)	4.777 (5.934)	35.642 (20.991)
Moss	0.006 (0.013)	0.001 (0.004)	0.019 (0.066)	0 (0)	0.878 (1.631)	0.322 (0.592)	0.312 (0.338)
Mud	0 (0)	0 (0)	0 (0)	0 (0)	0.314 (0.567)	0 (0)	152.29 (24.26)
Needles	0.001 (0.003)	0.004 (0.008)	0.024 (0.051)	0.032 (0.038)	0.008 (0.017)	3.922 (3.387)	0.04 (0.046)
Plant down	0.128 (0.177)	0 (0)	0 (0)	0 (0)	0 (0)	0 (0)	0.013 (0.025)
Rootlets	0.537 (0.549)	0.003 (0.005)	0.058 (0.128)	1.042 (0.535)	0.192 (0.237)	0.026 (0.047)	0.748 (0.777)
Shredded coarse stems	1.852 (0.995)	1.51 (0.677)	0.052 (0.186)	0.078 (0.092)	1.462 (2.527)	0.147 (0.121)	3.237 (3.936)
Sticks	0.006 (0.017)	0.001 (0.004)	0 (0)	0.025 (0.042)	29.227 (13.044)	0.743 (0.762)	4.157 (3.013)

*Note*: Presented are means and SDs in parentheses.

**TABLE A5:** Raw masses (g) of nest material in the interior section of the nest among our seven focal species.

	Prairie warbler	Chestnut‐sided warbler	Field sparrow	Chipping sparrow	Gray catbird	Eastern towhee	American robin
Sample size	9	7	13	6	13	9	4
Anthropogenic	0.112 (0.25)	0 (0)	0.008 (0.031)	0.022 (0.044)	0.01 (0.036)	0 (0)	0 (0)
Bark	0.001 (0.003)	0.001 (0.004)	0 (0)	0.008 (0.02)	2.967 (2.402)	0.244 (0.27)	0.078 (0.117)
Feathers	0.146 (0.224)	0 (0)	0.001 (0.003)	0.003 (0.005)	0.063 (0.218)	0 (0)	0.01 (0)
Fine stems	0.022 (0.035)	0.713 (0.296)	0.231 (0.274)	0.06 (0.051)	0.774 (0.904)	0.373 (0.804)	0.122 (0.238)
Flowers	0.004 (0.005)	0.004 (0.005)	0 (0)	0.002 (0.004)	0.016 (0.03)	0.019 (0.032)	0.022 (0.029)
Grasses and sedges	0.544 (0.329)	0.314 (0.395)	1.808 (0.716)	0.648 (0.399)	0.175 (0.289)	1.892 (1.637)	3.24 (1.976)
Hair	0.113 (0.203)	0.006 (0.005)	0.008 (0.014)	0.11 (0.106)	0.08 (0.282)	0 (0)	0 (0)
Intact coarse stems	0.129 (0.387)	0 (0)	0 (0)	0.002 (0.004)	0.121 (0.168)	0.124 (0.169)	0.128 (0.105)
Invertebrate silk and eggs	0.006 (0.005)	0.007 (0.008)	0 (0)	0 (0)	0 (0)	0 (0)	0 (0)
Leaves	0.01 (0.012)	0.006 (0.005)	0.001 (0.003)	0 (0)	3.15 (2.913)	0.606 (1.011)	0.085 (0.054)
Miscellaneous	0.302 (0.385)	0.014 (0.016)	0.068 (0.086)	0.062 (0.057)	1.273 (1.119)	0.742 (0.797)	1.857 (0.74)
Moss	0.011 (0.026)	0.003 (0.005)	0.124 (0.447)	0.008 (0.02)	0.074 (0.228)	0.744 (0.981)	0.032 (0.033)
Mud	0 (0)	0 (0)	0.003 (0.011)	0 (0)	0.248 (0.42)	0 (0)	1.183 (1.613)
Needles	0.001 (0.003)	0.001 (0.004)	0.005 (0.014)	0.002 (0.004)	0.389 (1.08)	1.682 (0.85)	0.035 (0.04)
Plant down	0.301 (0.264)	0 (0)	0 (0)	0 (0)	0.001 (0.003)	0 (0)	0 (0)
Rootlets	0.207 (0.224)	0.05 (0.104)	0.061 (0.193)	0.443 (0.37)	6.718 (3.333)	0.072 (0.213)	0.01 (0)
Shredded coarse stems	0.124 (0.321)	0.744 (1.014)	0 (0)	0.003 (0.008)	1.057 (2.293)	0.029 (0.049)	0.115 (0.122)
Sticks	0.001 (0.003)	0 (0)	0 (0)	0 (0)	1.003 (0.848)	0.026 (0.04)	0.195 (0.079)

*Note*: Presented are means and SDs in parentheses.

**TABLE A6:** Raw masses (g) of identified grass and sedge genera within the entire nest among our seven focal species.

Plant genus	Prairie warbler	Chestnut‐sided warbler	Field sparrow	Chipping sparrow	Gray catbird	Eastern towhee	American robin
Agrostis	0.349 (0.31)	0 (0)	0.051 (0.123)	0.342 (0.461)	0.002 (0.004)	0 (0)	0 (0)
Aristida	0.01 (0.028)	0 (0)	0.037 (0.081)	0.003 (0.008)	0 (0)	0.003 (0.008)	0 (0)
Bulbostylis	0.021 (0.042)	0 (0)	0.6 (0.737)	0.602 (0.582)	0 (0)	0.114 (0.302)	0 (0)
Carex	0 (0)	0.333 (0.577)	0.439 (0.825)	0.033 (0.082)	0.022 (0.053)	0.099 (0.152)	0.47 (0.94)
Danthonia	0 (0)	0 (0)	0.001 (0.003)	0 (0)	0 (0)	0 (0)	0 (0)
Dichanthelium	0.048 (0.077)	0.087 (0.15)	1.443 (1.514)	0.112 (0.133)	0.077 (0.08)	0.567 (0.736)	2.953 (3.682)
Digitaria	0.006 (0.012)	0 (0)	0.007 (0.009)	0.027 (0.038)	0 (0)	0.347 (0.884)	0 (0)
Eragrostis	0.005 (0.014)	0 (0)	0 (0)	0.002 (0.004)	0 (0)	0 (0)	0 (0)
Juncus	0 (0)	0 (0)	0.008 (0.018)	0.002 (0.004)	0.002 (0.004)	0.131 (0.343)	0.013 (0.025)
Muhlenbergia	0 (0)	0.09 (0.156)	0 (0)	0 (0)	0 (0)	0 (0)	0 (0)
Schizachyrium	0 (0)	0 (0)	0.007 (0.023)	0 (0)	0 (0)	0 (0)	0 (0)

*Note*: Presented are means and SDs in parentheses.
